# Impact of barriers and motivators on intention and confidence to undergo hereditary cancer genetic testing

**DOI:** 10.1002/jgc4.1926

**Published:** 2024-05-27

**Authors:** Sarah Austin, Erika N. Hanson, Emerson Delacroix, Elizabeth Bacon, John Rice, Lynette Hammond Gerido, Elizabeth Rizzo, Versha Pleasant, Elena M. Stoffel, Jennifer J. Griggs, Ken Resnicow

**Affiliations:** ^1^ Rogel Cancer Center University of Michigan Ann Arbor Michigan USA; ^2^ Department of Internal Medicine University of Michigan School of Medicine Ann Arbor Michigan USA; ^3^ University of Michigan School of Public Health Ann Arbor Michigan USA; ^4^ Case Western Reserve University School of Medicine Cleveland Ohio USA

**Keywords:** barriers, genetic testing, health behavior, intention, knowledge

## Abstract

Genetic testing for hereditary cancer syndromes can provide lifesaving information allowing for individualized cancer screening, prevention, and treatment. However, the determinants, both barriers and motivators, of genetic testing intention are not well described. A survey of barriers and motivators to genetic testing was emailed to adult patients eligible for genetic testing based on cancer diagnosis who previously have not had genetic testing (*n* = 201). Associations between barriers/motivators with testing intention and confidence were examined first by correlation followed by multivariable linear regression model holding constant potential covariates. Seven barrier items from two domains (logistics and genetic testing knowledge) were found to significantly negatively correlate with genetic testing intention. Unexpectedly, three barrier items had significant positive correlation with genetic testing intention; these were related to family worry (passing a condition on to future generations) and testing knowledge (needing more information on the genetic testing process and what it has to offer). Ten barrier items had significant negative correlation with confidence to get a genetic test and encompassed four domains: stigma, insurance/genetic discrimination, knowledge, and cost. All motivator items were associated with intention to get a genetic test, while none were associated with confidence. Multivariable analysis yielded six total barriers (five from the knowledge domain, one from cost domain) and two motivators (relieved to know and treatment impact) that were significantly associated with genetic testing intention or confidence when controlling for demographic characteristics. These findings indicate the need for tailored interventions to amplify motivating factors and counter‐message barriers to enhance patient motivation and confidence to undergo testing.

**What is known about this topic**

Numerous barriers and motivators to genetic testing have been identified, but there is limited evidence indicating which are more salient and should be incorporated into clinical practice.

**What this paper adds to the topic**

Multivariable analysis identified a list of barriers primarily related to genetic testing knowledge and cost, as well as motivators including feeling relieved and impact on cancer treatment as potential items necessitating intervention for patients across multiple cancer types, including a large proportion of patients with prostate cancer. Some items previously described as barriers to testing, including worry about passing a gene to future generations and needing more information on genetic testing, were found to be motivating factors for patients.

## 1 INTRODUCTION

Genetic testing for hereditary cancer syndromes is a bridge to targeted cancer screening, prevention, and treatment. While genetic risk assessment and testing is a powerful medical tool, individual and healthcare system level barriers prevent many eligible patients from undergoing genetic testing (Childers et al., 2017; Clark et al., 2023; Ladd et al., 2020; Turza et al., 2022; Walker et al., 2019). For example, fewer than one in five patients with breast or ovarian cancer eligible for *BRCA1/BRCA2* testing have undergone genetic testing (Childers et al., 2017). Uptake is similar or lower amongst patients with other cancer types including pancreatic and prostate cancer, despite recommendation from national guidelines, with one institution reporting less than 40% of eligible patients with prostate cancer having been referred for genetic testing and only 10.5% completing testing (Daly et al., 2021; Suri et al., 2022; Walker et al., 2019).

Several patient‐level barriers to genetic testing exist and have been previously described. These include limited knowledge about the utility of testing, psychosocial concerns such as fear and anxiety related to coping with test results, and concerns about privacy and confidentiality of their genetic information (Chang et al., 2022; Halverson et al., 2020; Loeb et al., 2021; Mallen et al., 2021; Morand et al., 2022; Shaw et al., 2018; Smith‐Uffen et al., 2021; Sweeny et al., 2014; Vogel et al., 2018). Other barriers fall in the domain of logistics. These include transportation to appointments, proximate access to testing clinics, appointment scheduling, and cost/insurance coverage of testing. These systemic issues are highlighted by Loeb et al., in which providers report several themes impacting genetic testing uptake for patients with prostate cancer including variability in provider knowledge on guidelines and referral practices, time and resources available to coordinate testing, and both patient and provider concerns with test coverage (Loeb et al., 2021). These barriers may be especially daunting for individuals navigating a recent cancer diagnosis and prevent some from completing testing, even if motivated (Chang et al., 2022; Geer et al., 2001; Loeb et al., 2021; Mallen et al., 2021; Morand et al., 2022; Smith‐Uffen et al., 2021).

Studies have also identified motivating factors, or drivers, to obtain genetic testing, such as wanting to provide information to family members, potential for improved treatment and surveillance options, and provider recommendation to undergo testing (Chang et al., 2022; Gomez‐Trillos et al., 2020; Ladd et al., 2020; Loeb et al., 2021; Mallen et al., 2021; Scott et al., 2020; Smith‐Uffen et al., 2021). Yet, research is limited about the relationship between these perceived barriers/motivators and intention to pursue genetic testing.

Kessler et al. (2005) found that for women with a moderate or high risk of having a *BRCA1/BRCA2* pathogenic variant, beliefs about cancer screening, number of family members with cancer, and perception of one's own of *BRCA1/BRCA2* risk were positive predictors of genetic testing intention (Kessler et al., 2005). Similarly, Durfy et al. (1999) surveyed women with a family history of breast cancer and found that their perceived risk of developing breast cancer, current level of breast cancer worry, and access to testing were positive predictors of genetic testing interest, while stigma associated with testing was a negative predictor of genetic testing interest (Durfy et al., 1999). These studies were conducted exclusively in patients with a personal/family history of breast and ovarian cancer and were conducted more than 20 years ago when testing availability, coverage, and guidelines were more limited. A more recent study interviewed Latina women who met genetic testing criteria for hereditary breast and ovarian cancer to identify barriers, facilitators, and perceptions of genetic risk assessment. Despite many participants reporting limited awareness of genetic services, knowledge gaps related to cancer and hereditary risk, and barriers navigating the healthcare system in general, respondents reported interest in pursuing genetic testing and were motivated to inform treatment decisions and their family members (Gomez‐Trillos et al., 2020).

A broader understanding of how barriers and motivators impact genetic testing decisions across multiple cancer types is needed. The aim of this study is to examine how barriers and motivators relate to intention and confidence to undergo hereditary cancer genetic testing amongst patients who had not been previously tested.

## 2 METHODS

### 2.1 Participants

Inclusion criteria were patients over age 18, who completed an inpatient or outpatient encounter with any clinician at Michigan Medicine (a large academic medical center based in Ann Arbor, Michigan) between 1 Jan 2019–15 Feb 2021, with a documented ICD‐9 or ICD‐10 code indicating a diagnosis of breast, prostate, colorectal, endometrial, ovarian, or pancreatic cancer in their electronic medical record. Patients with breast cancer were included if they were diagnosed under the age of 50. Patients with prostate cancer were included if they were diagnosed under 50 or had metastatic disease. Patients with colon or rectal, endometrial, ovarian, or pancreatic cancer were eligible regardless of age at diagnosis. This inclusion criterion was created based on a modified version of current (at enrollment) national guidelines for genetic testing uptake (Daly et al., 2021; Weiss et al., 2021). Participants could report more than one cancer type. The University of Michigan Medical School Institutional Review Board approved contacting patients who met the above criteria with application HUM0019157.

### 2.2 Procedures

We conducted a cross‐sectional online survey to understand barriers and motivators for genetic testing intention. An email invitation to a Qualtrics^XM^ survey was sent to eligible patients. No reminders were sent. Those who completed the digital consent were enrolled and offered $10 for survey completion. Initially, eligible patients were invited regardless of their genetic testing status. However, after receiving 532 completed surveys, those who reported previously undergoing genetic testing (*N* = 410) were overrepresented in our sample population (44 did not answer, 78 did not have genetic testing). To balance the number of untested individuals, a screener question was later added to exclude tested individuals. A large percentage of the untested individuals had a history of prostate cancer. This led to the addition of 165 untested participants in this phase of recruitment (*N* = 243 untested respondents overall).

### 2.3 Instrumentation

Through a literature review and discussions with subject area experts we identified 26 unique barriers and 9 unique motivators to testing that were included in the survey (Fogel et al., 2017; Fogleman et al., 2019; Gill et al., 2020; Hafertepen et al., 2017; Hurtado‐de‐Mendoza et al., 2018; O'Neill et al., 2013; Paller et al., 2019; Shaw et al., 2018). We created a common response scale for the 35 items which were answered along a 5‐point continuum ranging from Not at all true (1) to Very true (5). Barrier and motivator items were grouped into conceptual domains created by the authors and were not grouped based on statistical analysis. Barrier item domains include family worry, cost, knowledge, insurance/genetic discrimination, religious/spiritual beliefs, logistics, and stigma. Motivator item domains include actionability and emotions.

Participants were asked to respond to three items related to genetic testing intention using a 10‐point Likert scale (0 = not likely, 10 = extremely likely): (1) How ready are you to get genetic testing in the next year? (2) How confident are you that you can get genetic testing in the next year? (3) How important to you is getting genetic testing in the next year? Factor analysis showed that readiness and importance (items 1 and 3) aggregated to form a single factor, defined as intention score (average of readiness and importance). Alpha in this sample was 0.86. Confidence was left as a single item.

Descriptive variables collected included sex assigned at birth, gender identity (dichotomized into “male” or “female”), current age, education (collapsed into four categories), income (collapsed into three categories), insurance status (dichotomized into public [Medicare, Medicaid, Tri‐care, Veterans Affairs, Indian Health Service] and private [employer‐funded]), employment status, race/ethnicity, and cancer type (prostate vs other).

### 2.4 Data analysis

Demographic variables and cancer type were summarized by frequency. Descriptive statistics (mean and standard deviation) were calculated for intention score and confidence across all demographic variables. Associations between barriers/motivators with testing intention and confidence were calculated with Pearson correlations.

A general linear regression model was used to investigate the relationship between each barrier/motivator item (independent variable) with intention and confidence (dependent variable). Significant items from the univariate analyses (those with *p* ≤ 0.05) were included in the final regression model. This model controlled demographic variables previously described as factors impacting genetic testing uptake and/or intention, including age, education (dummy coded with post‐secondary as reference), sex, cancer type (prostate vs. other), insurance type (public vs. private), race (dummy coded with white race as the reference), employment status (employed vs. unemployed), and income ($30,000–$74,999 as reference; Chang et al., 2022; Clark et al., 2023; Fogleman et al., 2019; Manrriquez et al., 2018; Wehbe et al., 2022). We also tested whether the association of any significant barrier or motivator with intention and confidence was moderated by cancer type using an indicator variable of prostate vs all other cancer types.

Data were analyzed using SPSS v.28. The dataset supporting this study is available at https://doi.org/10.7302/59xr‐c178.

## 3 RESULTS

### 3.1 Demographics

787 out of 3000 (26.2%) invitees responded to the survey, of whom 243 reported no prior genetic testing and answered barrier/motivator questions. 42 respondents were ineligible based on cancer types/age at diagnosis as they had incorrect ICD‐10 codes in their chart. Therefore, 201 respondents were included in our analysis. The majority were male gender (70.1%) with a diagnosis of prostate cancer (65.2%). The remaining cancer type distribution included ovarian (11.9%), breast (10.0%), pancreatic (8.0%), colorectal (5.9%) and uterine cancer (5.0%) (data not shown). Most patients were white (89.6%), over the age of 65 (61.2%), with an advanced degree (46.3%). A description of the study sample and mean/standard deviation of intention score and confidence across demographic variables is found in Table 1.

**TABLE 1:** Descriptive statistics of survey participants (*n* = 201).

Demographics	*N*	%	Mean intention (SD)	Mean confidence (SD)
Age				
35 and under	1	1.0%	3.00 (0.71)	5.50 (3.53)
36–50	26	12.9%	5.65 (3.63)	4.69 (3.75)
51–64	50	24.9%	5.44 (3.17)	5.34 (2.82)
65+	123	61.2%	4.75 (3.04)	4.67 (3.33)
Gender				
Male	141	70.1%	5.01 (3.55)	4.74 (3.49)
Female	58	28.9%	5.03 (3.01)	4.88 (3.18)
Race				
White	180	89.6%	4.89 (3.11)	4.88 (3.22)
Black	8	4.0%	5.06 (2.84)	5.50 (3.62)
Multi‐racial	3	2.0%	8.25 (1.55)	4.50 (5.26)
Hispanic	3	1.5%	6.83 (3.78)	3.33 (4.16)
Middle Eastern and North African	2	1.0%	5.00 (7.07)	6.00 (1.41)
Other (includes American Indian, Asian, other)	4	2.0%	6.25 (4.29)	3.00 (2.94)
Income				
Under $30,000	14	7.0%	6.28 (3.50)	5.57 (3.86)
$30,000–$74,999	55	27.4%	4.75 (3.06)	4.78 (3.08)
$75,000 and above	105	52.2%	4.92 (3.11)	5.06 (3.29)
Education				
None through high school/GED	5	2.5%	5.40 (3.56)	4.80 (1.64)
Post‐secondary (trade school/some college/associates)	43	21.4%	5.79 (3.21)	4.47 (3.12)
Bachelors	59	29.4%	4.53 (3.17)	4.61 (3.38)
Advanced degree (Masters, Doctoral/Professional)	93	46.3%	4.97 (3.08)	5.05 (3.35)
Employment				
Full‐time work	57	28.4%	5.20 (3.29)	5.23 (2.99)
Part‐time work	24	11.9%	5.21 (3.36)	5.67 (3.67)
Student	2	1.0%	6.00 (1.41)	0.00 (0.00)
Out of work	14	7.0%	6.36 (3.09)	4.86 (3.88)
Retired	104	51.7%	4.68 (3.04)	4.55 (3.17)
Insurance				
Public or government insurance	112	55.7%	4.83 (3.08)	4.44 (3.37)
Private insurance	85	42.3%	5.15 (3.22)	5.41 (3.04)
Other source of insurance	3	1.5%	7.00 (3.00)	6.00 (2.65)
Cancer type^a^				
Prostate	131	65.2%	5.00 (2.97)	4.98 (3.18)
Other	70	34.8%	5.05 (3.48)	4.60 (3.40)

Outcome variable mean	Mean	SD	Range
Confidence	4.85	3.26	10
Intention	5.02	3.15	10

^a^ Patients could report multiple primary cancers.

### 3.2 Univariate analyses

#### 3.2.1 Barrier associations with genetic testing intention

Correlations between barriers and genetic testing intention and confidence are shown in Table 2. Seven barrier items had significant negative correlation with genetic testing intention. These items were from two domains: logistics (timing) and knowledge (no purpose, GT accuracy, cancer risk/recurrence fear, cancer prevention, unknown benefit, cancer screening). Three putative barrier items, unexpectedly, had significant positive correlation with genetic testing intention. These items were from two domains: family worry (inheritance worry) and knowledge (more info and unclear goals).

**TABLE 2:** Pearson correlations of barriers to genetic testing intention and confidence in untested respondents (*n* = 201).

Barrier item	Correlation with intention	*p*	Correlation with confidence	*p*
Domain: Family worry				
Feeling guilty if one of my relatives had an altered gene and I did not (inheritance guilt)	−0.02	0.808	−0.02	0.733
Feeling guilty if my family member developed cancer (survivor guilt)	−0.10	0.185	−0.04	0.566
Worry more about family members who could be carriers (family worry)	−0.04	0.586	0.07	0.315
Worry about passing the gene to future generations (inheritance worry)	0.21**	0.003	0.27**	<0.001
Domain: Cost				
Getting GT would be too expensive (GT cost)	0.04	0.571	−0.29**	<0.001
Health insurance would not cover the cost of genetic testing (GT insurance coverage)	0.11	0.134	−0.23**	<0.001
Domain: Knowledge				
No purpose in testing if someone already has cancer (no purpose)	−0.36**	<0.001	−0.15*	0.039
Not sure if the test is accurate (GT accuracy)	−0.28**	<0.001	−0.26**	<0.001
GT would not help me deal with my fears and uncertainty about having my cancer come back or getting a new one (cancer risk/recurrence fear)	−0.29**	<0.001	−0.17*	0.014
GT results would not help prevent cancer (cancer prevention)	−0.26**	<0.001	−0.24**	<0.001
Unknown benefit (unknown benefit)	−0.21**	0.003	−0.11	0.128
GT results would not impact my cancer screening/follow‐up (cancer screening)	−0.16*	0.026	−0.06	0.403
Don't know how to get GT (how to get GT)	0.02	0.730	−0.27**	<0.001
Need more information on GT process (more info)	0.15*	0.033	−0.14	0.052
Need to get more information about what genetic testing has to offer (unclear goals)	0.29**	<0.001	−0.02	0.805
Domain: Insurance/genetic discrimination				
Worry about who would have access to my test results (GT privacy)	−0.12	0.093	−0.12	0.132
Worry about how GT would affect my health insurance (insurance coverage worry)	−0.11	0.127	−0.15*	0.030
Worry about pre‐existing condition for health insurance (insurance discrimination worry)	−0.14	0.056	−0.15*	0.032
If I were found to carry an altered gene, I worry it would affect my life insurance policy (life insurance worry)	−0.10	0.145	−0.03	0.698
I would worry about how GT results affect my employment (employment worry)	−0.03	0.663	−0.12	0.095
Domain: Religious/spiritual beliefs				
Getting GT is not consistent with my religious or spiritual beliefs (religion/spiritual)	−0.05	0.453	−0.09	0.199
Domain: Logistics				
Too much going on to think about genetic testing right now (timing)	−0.17*	0.018	−0.05	0.460
Domain: Stigma				
I am concerned about my family's reaction to my GT results (family reaction)	−0.09	0.221	−0.10	0.171
Having a gene mutation would cause others to view me negatively (negative perception)	−0.04	0.547	−0.14	0.052
Having a gene mutation would make me feel singled out (isolation)	−0.13	0.079	−0.08	0.244
I am concerned about my partner's reaction to my GT results (partner reaction)	−0.09	0.202	−0.14*	0.045

Abbreviation: GT, genetic testing.

* *p* < 0.05.

** *p* < 0.01.

#### 3.2.2 Barrier associations with genetic testing confidence

Ten barriers had significant negative correlation with confidence to get a genetic test and included four domains: stigma, insurance/genetic discrimination, knowledge, and cost (Table 2). One barrier item had a significant positive correlation with confidence: inheritance worry. Four barriers had a significant negative correlation with both genetic testing intention and confidence. These were all from the knowledge domain: no purpose, GT accuracy, cancer risk/recurrence fear, and cancer prevention.

#### 3.2.3 Motivator correlations

Correlations between motivator items and genetic testing intention and confidence are found in Table 3. All nine motivator items were significantly positively associated with genetic testing intention (*p* < 0.01). None of the motivator items had statistically significant correlations with genetic testing confidence.

**TABLE 3:** Pearson correlations of motivators to genetic testing intention and confidence in untested respondents (*n* = 201).

Motivator items	Correlation with intention	*p*	Correlation with confidence	*p*
Domain: Actionability				
Help to plan treatment (treatment impact)	0.39**	<0.001	0.07	0.348
Increase my sense of personal control (autonomy)	0.37**	<0.001	0.05	0.452
Impact decisions on cancer screening (screening impact)	0.26**	<0.001	0.05	0.502
Domain: Emotions				
Empowered	0.22**	0.002	−0.02	0.793
Prepared	0.25**	<0.001	0.04	0.605
Knowledgeable	0.23**	0.001	0.05	0.499
Responsible	0.21**	0.004	0.002	0.976
Purposeful	0.36**	<0.001	0.03	0.695
Relieved to know	0.35**	<0.001	0.03	0.722

** *p* < 0.01.

### 3.3 Multivariable analyses

#### 3.3.1 Predictors of genetic testing intention

In regression analyses three barrier items (no purpose, cancer risk/recurrence fear, GT accuracy) and two motivator items (relieved to know and treatment impact) were found to be significantly associated with genetic testing intention when controlling for age, education, gender, cancer type, health insurance, income, race, and employment status (Table 4). The *R* ^2^ of the model is 0.370 (*p* < 0.001).

**TABLE 4:** Predictors of genetic testing intention from multivariable regression analysis.

Independent variable	*t*	*p*	Unstandardized *B* (standard error)	Standardized Beta coefficient
Relieved to know	2.56	0.011	0.45 (0.17)	0.18
Help to plan treatment (treatment impact)	2.45	0.015	0.48 (0.20)	0.18
No purpose in testing if someone already has cancer (no purpose)	−2.41	0.017	−0.50 (0.21)	−0.17
GT would not help me deal with my fears and uncertainty about having my cancer come back or getting a new one (cancer risk/recurrence fear)	−3.61	<0.001	−0.53 (0.15)	−0.22
Not sure if the test is accurate (GT accuracy)	−2.33	0.02	−0.43 (0.18)	−0.15

*R*	0.608
*R* ^2^	0.370
Adjusted *R* ^2^	0.316
*F*_16,187_	6.85, *p* < 0.001

*Note*: Model included additional barrier items: cancer prevention, cancer screening, timing, and motivator items: autonomy, screening impact, empowered, prepared, knowledgeable, responsible, purposeful. Controlled for age, gender, cancer type, insurance, education, race, income, and employment status.

Abbreviations: GT, genetic testing; N/A, not applicable.

#### 3.3.2 Predictors of genetic testing confidence

Three barrier items (GT cost, cancer prevention, how to get GT) were found to be significant predictors of genetic testing confidence, when controlling for age, education, gender, cancer type, health insurance, income, race, and employment status (Table 5). The *R* ^2^ of the model is 0.228 (*p* < 0.001). We found no moderating effect of prostate vs other types of cancer for any of the significant barriers or motivators for intention or confidence. The effect size of these associations is in the small to moderate range (Gignac & Szodorai, 2016).

**TABLE 5:** Predictors of genetic testing confidence from multivariable regression analysis.

Independent variable	*t*	*p*	Unstandardized *B* (standard error)	Standardized Beta coefficient
Getting GT would be too expensive (GT cost)	−3.66	<0.001	−0.78 (0.21)	−0.27
GT results would not help prevent cancer (cancer prevention)	−2.76	0.006	−0.51 (0.19)	−0.19
Don't know how to get GT (how to get GT)	−2.21	0.03	−0.37 (0.17)	−0.15

*R*	0.477
*R* ^2^	0.228
Adjusted *R* ^2^	0.170
*F*_14,189_	3.98, *p* < 0.001

*Note*: Model included additional barrier items: cancer risk/recurrence fear, genetic testing accuracy, genetic testing insurance coverage, insurance coverage worry, insurance discrimination worry, partner reaction, and no purpose. Controlled for age, gender, cancer type, insurance, education, race, income, and employment status.

Abbreviations: GT, genetic testing; N/A, not applicable.

## 4 DISCUSSION

In this cross‐sectional study, we examined the association between a wide range of barriers and motivators for cancer genetic testing across multiple cancer types using two distinct outcomes: intention and confidence. In univariate analysis barriers related to genetic testing knowledge had the strongest significant negative association with intention to undergo genetic testing for hereditary cancer risk, followed by logistic barriers. Similarly, barriers related to knowledge as well as cost, stigma, and insurance/genetic discrimination were significantly negatively associated with confidence to undergo genetic testing for hereditary cancer risk. All motivator items were significantly associated with genetic testing intention, whereas no motivator items were significantly associated with genetic testing confidence. This is expected, given that motivation to pursue genetic testing does not account for the perceived difficulty or efficacy in completing that action (e.g., confidence).

In multivariable analysis three barrier items, all from the knowledge domain (no purpose, cancer risk/recurrence fear, GT accuracy), and two motivators (relieved to know [domain: emotions] and treatment impact [domain: actionability]) were significantly associated with genetic testing intention. Three barriers were significantly associated with confidence: GT cost (domain: cost), cancer prevention (domain: knowledge), and how to get GT (domain: knowledge). In sum, multivariable analysis yielded six total barriers (five of which were from the knowledge domain) and two motivators that were significantly associated to genetic testing intention or confidence.

Our findings regarding low perceived knowledge as a barrier to intention and confidence are consistent with findings from previous qualitative studies. Kne et al. (2017) completed focus groups for women at high risk for breast and ovarian cancer and found that limited knowledge was a factor that contributed to their decisions not to pursue testing. Similarly, Mallen et al. (2021) interviewed patients with ovarian cancer and their genetics providers to explore experiences with genetic testing; all participants reported patient knowledge, specifically lack of knowledge about the existence of and relationship between genetic testing and cancer, as a barrier to pursuing genetic counseling and testing.

Our identified barriers from the knowledge and cost domain are also substantiated by Hayden et al. (2017) who found that patient disinterest, which encompassed knowledge gaps including “patient sees no benefit”, and cost/insurance concerns were reasons patients undergoing genetic counseling for *BRCA*‐related cancers decided to decline genetic testing. In our study, these knowledge barriers were found to be predictive in a sample of patients with multiple cancer types, including male patients with prostate cancer and those with cancers outside of the *BRCA*‐associated cancer spectrum. Identification of these barriers is not only important for identifying gaps in patient care, but also for tailored intervention.

Some putative barrier items in univariate analysis were unexpectedly positively associated with intention and confidence to undergo testing, despite previous literature suggesting a negative association (Smith‐Uffen et al., 2021). For example, we found that worry about passing on a gene to future generations operated more as a motivator for both confidence and intention than a barrier. Concern for family members has been reported by patients as both a barrier and motivating factor for testing, suggesting that despite this worry, patients feel driven to undergo testing to provide information to relatives (Smith‐Uffen et al., 2021). To evaluate this items role as a barrier in future assessments, it may be helpful to re‐word as follows: “A reason I don't get tested is because I don't want to have to share difficult news with my family” to better connect sharing information as being the barrier to genetic testing. Other “barriers”, such as needing more information on the genetic testing process, or what genetic testing has to offer, may indicate interest in getting testing rather than concern or skepticism.

### 4.1 Practice implications

Together our findings indicate the need for interventions to amplify motivating factors and counter‐message barriers to enhance patient motivation and confidence to undergo genetic testing for hereditary cancer risk. Psychoeducational interventions can be completed through both group level intervention and individually tailored messaging (Gerido et al., 2023). Counter‐messaging for barriers related to utility, accuracy, and cost could include providing key facts dispelling genetic testing misconceptions, examples of how knowing one's genetic status may impact medical management (ex: more frequent colonoscopies, consideration of chemoprevention medication), and information related to general out of pocket cost for genetic testing. Enhancing motivators could include helping individuals find personal meaning in undergoing genetic testing and sharing results with family. In particular, interventions could align getting genetic testing and sharing results with relatives with values such as protecting family and serving as a role model (Gerido et al., 2023). This messaging can be communicated through various modalities including traditional genetic counseling, patient advocates, community health workers, educational videos, digital tools such as apps or chat bots and decision aids to improve pretest education for patients (Fournier et al., 2018; McCuaig et al., 2018; Raspa et al., 2021; Russo et al., 2021). Digital tools are particularly important given the shortage of genetic counselors and increased demand for genetic counseling services.

It may be beneficial for the genetic counseling profession to utilize a standard set of barriers and motivators as part of routine pretest counseling, perhaps completed through patient portal or counselor interviewing. Knowing individual level barriers and motivators can help the genetic counselor tailor their session to the needs of the patient or in some cases, when barriers are low and motivators are high, the patient could proceed to testing without a motivational session through their clinician, and instead through point of care options such as a genetic testing station or patient‐initiated testing (Walker et al., 2021). This is especially important as the expansion to alternative service delivery models is required to meet the increasing demand for hereditary cancer genetic testing.

### 4.2 Limitations

The study has several limitations. First, the study was based on a single academic healthcare systems cancer registry. Our sample was not racially diverse and included predominately patients with prostate cancer. Whether findings can be generalized to more diverse clinical populations and settings needs to be further examined. Despite effort to compile a comprehensive set of motivator and barrier items, it is possible there could be missed or novel items not included in the study, or the barrier and motivator items may not hold for other groups. Genetic testing status was self‐reported and may not be accurate of testing status. Our study only measured intention and confidence to undergo genetic testing, not actual uptake of genetic testing. This study did not assess if genetic testing was previously offered to patients and did not assess which providers offered the testing. Since our study was cross‐sectional in design, longitudinal prediction of testing behavior is not possible and longitudinal studies to examine causal pathways are needed. As part of a larger clinical trial, we plan to assess the relationship between patient reported barriers and motivators to testing with actual genetic testing uptake for individuals who have not previously undergone genetic testing. Finally, we only included patients that were eligible for genetic testing based on their personal history of cancer. Additional individuals may qualify for genetic testing with the addition of family history and were not included in this study.

### 4.3 Future research

Our future studies include assessing barriers and motivators, as well as genetic testing uptake, from patients across multiple health systems in Michigan as part of a larger clinical trial. This work could elucidate the level to which motivator and barrier beliefs ultimately predict uptake, moving beyond the current paper which only includes intention.

## AUTHOR CONTRIBUTIONS

Author, Sarah Austin, confirms that they had full access to all the data in the study and take responsibility for the integrity of the data and the accuracy of the data analysis. All of the authors gave final approval of this version to be published and agree to be accountable for all aspects of the work in ensuring that questions related to the accuracy or integrity of any part of the work are appropriately investigated and resolved.

## FUNDING INFORMATION

National Cancer Institute, Grant/Award Number: U01 CA232827‐05. Innovative Approaches to Expand Cancer Genetic Screening and Testing for Patients & Families in a Statewide Oncology Network through Community, State, & Payer Partnerships (PI Stoffel, Griggs, Resnicow).

## CONFLICT OF INTEREST STATEMENT

Authors Sarah Austin, Erika N. Hanson, Emerson Delacroix, Elizabeth Bacon, Lynette Hammond Gerido, Elizabeth Rizzo, Versha Pleasant, Elena M. Stoffel, Jennifer J. Griggs, and Ken Resnicow declare that they have no conflict of interest.

## ETHICS STATEMENT

Human studies and informed consent: The University of Michigan Medical School Institutional Review Board (IRB) approved this study, which involved contacting patients who met the eligibility criteria via email (HUM0019157). Potential participants were presented with a consent and waiver of formal written consent was granted by the IRB.

Animal studies: No non‐human animal studies were carried out by the authors for this article.

## Supporting information


Data S1.

## Data Availability

The dataset supporting this study is available at https://doi.org/10.7302/59xr‐c178.
