# Supplementary material for: Impact of barriers and motivators on intention and confidence to undergo hereditary cancer genetic testing
Source: J Genet Couns. 2024 May 27;34(1):e1926. doi: 10.1002/jgc4.1926 (PMC11599461; doi:10.1002/jgc4.1926)
Supplement: Supplementary file 1 — Data S1. [file JGC4-34-0-s001.docx]

| **Please tell us how true each of these is for you.** | Not at all true (1) | | | Somewhat untrue (2) | | | Neither true nor untrue (3) | | | Somewhat true (4) | | | Very true (5) | | |
| --- | --- | --- | --- | --- | --- | --- | --- | --- | --- | --- | --- | --- | --- | --- | --- |
| I need to get more information about what genetic testing has to offer. |  | | |  | | |  | | |  | | |  | | |
| I don’t know how genetic testing benefits me. |  | | |  | | |  | | |  | | |  | | |
| I need more information about the how the testing process works. |  | | |  | | |  | | |  | | |  | | |
| I don’t know how to get genetic testing. |  | | |  | | |  | | |  | | |  | | |
| Genetic testing would not help me deal with my fears and uncertainty about having my cancer come back or getting a new one. |  | | |  | | |  | | |  | | |  | | |
| Genetic testing would not provide me with any means of preventing cancer. |  | | |  | | |  | | |  | | |  | | |
| I’m not sure if the test is accurate. |  | | |  | | |  | | |  | | |  | | |
|  | | Not at all true (1) | | | Somewhat untrue (2) | | | Neither true nor untrue (3) | | | Somewhat true (4) | | | Very true (5) | |
| Knowing my genetic status wouldn't impact my cancer screening/follow-up monitoring schedule. | |  | | |  | | |  | | |  | | |  | |
| Getting genetic testing would be too expensive for me. | |  | | |  | | |  | | |  | | |  | |
| I worry that my health insurance would not cover the cost of genetic testing. | |  | | |  | | |  | | |  | | |  | |
| I have too much going on with my current cancer diagnosis to think about genetic testing right now. | |  | | |  | | |  | | |  | | |  | |
| If I were found to carry an altered gene, I would worry about who would have access to my test results. | |  | | |  | | |  | | |  | | |  | |
| I would worry about how genetic testing would affect my health insurance. | |  | | |  | | |  | | |  | | |  | |
| If I were found to carry an altered gene, I worry it would be considered a pre-existing condition for health insurance. | |  | | |  | | |  | | |  | | |  | |
| If I were found to carry an altered gene, I worry it would affect my life insurance policy. | |  | | |  | | |  | | |  | | |  | |
| I would worry about how these results affect my employment. | |  | | |  | | |  | | |  | | |  | |
|  | | | Not at all true (1) | | | Somewhat untrue (2) | | | Neither true nor untrue (3) | | | Somewhat true (4) | | | Very true (5) |
| If I were found to carry an altered gene, I would worry about passing the gene to future generations. | | |  | | |  | | |  | | |  | | |  |
| Knowing that I carry an altered gene would cause me to worry more about other family members who could be carriers. | | |  | | |  | | |  | | |  | | |  |
| If I were found to carry an altered gene, I would feel guilty if my family member developed cancer. | | |  | | |  | | |  | | |  | | |  |
| If I were found to carry an altered gene, it would cause others to view me negatively. | | |  | | |  | | |  | | |  | | |  |
| If I were found to carry an altered gene, I would feel singled out. | | |  | | |  | | |  | | |  | | |  |
| I am concerned about my partner’s reaction to my genetic testing results. | | |  | | |  | | |  | | |  | | |  |
| I am concerned about my family’s reaction to my genetic testing results. | | |  | | |  | | |  | | |  | | |  |

|  | Not at all true (1) | Somewhat untrue (2) | Neither true nor untrue (3) | Somewhat true (4) | Very true (5) |
| --- | --- | --- | --- | --- | --- |
| I would feel guilty if one of my relatives had an altered gene and I did not. |  |  |  |  |  |
| Getting genetic testing is not consistent with my religious or spiritual beliefs. |  |  |  |  |  |
| I believe that if someone is diagnosed with cancer there is no purpose for genetic testing at that point. |  |  |  |  |  |

 **Please tell us how true each of these is for you.**

|  | Not at all true (1) | Somewhat untrue (2) | Neither true nor untrue (3) | Somewhat true (4) | Very true (5) |
| --- | --- | --- | --- | --- | --- |
| Knowing my genetic status could help me plan my treatment. |  |  |  |  |  |
| Knowing whether or not I carry an altered gene would increase my sense of personal control. |  |  |  |  |  |
| Knowing that I carry an altered gene would help me decide whether to go for more frequent cancer screening. |  |  |  |  |  |

If I was found to carry an altered gene, I would feel...

|  | Not at all true (1) | Somewhat untrue (2) | Neither true nor untrue (3) | Somewhat true (4) | Very true (5) |
| --- | --- | --- | --- | --- | --- |
| ...Empowered |  |  |  |  |  |
| ...Prepared |  |  |  |  |  |
| ...Knowledgeable |  |  |  |  |  |
| ...Responsible |  |  |  |  |  |
| ...Purposeful |  |  |  |  |  |
| ...Relieved to know |  |  |  |  |  |
